# Numerical Affordance Influences Action Execution: A Kinematic Study of Finger Movement

**DOI:** 10.3389/fpsyg.2018.00637

**Published:** 2018-05-01

**Authors:** Rosa Rugani, Sonia Betti, Luisa Sartori

**Affiliations:** ^1^Department of General Psychology, University of Padua, Padua, Italy; ^2^Padova Neuroscience Center, University of Padua, Padua, Italy

**Keywords:** mental number line, spatial-numerical association, kinematics, reaching, action execution, finger movement, numerical cognition

## Abstract

Humans represent symbolic numbers as oriented from left to right: the mental number line (MNL). Up to now, scientific studies have mainly investigated the MNL by means of response times. However, the existing knowledge on the MNL can be advantaged by studies on motor patterns while responding to a number. Cognitive representations, in fact, cannot be fully understood without considering their impact on actions. Here we investigated whether a motor response can be influenced by number processing. Participants seated in front of a little soccer goal. On each trial they were visually presented with a numerical (2, 5, 8) or a non-numerical ($) stimulus. They were instructed to kick a small ball with their right index toward a frontal soccer goal as soon as a stimulus appeared on a screen. However, they had to refrain from kicking when number five was presented (no-go signal). Our main finding is that performing a kicking action after observation of the larger digit proved to be more efficient: the trajectory path was shorter and lower on the surface, velocity peak was anticipated. The smaller number, instead, specifically altered the temporal and spatial aspects of trajectories, leading to more prolonged left deviations. This is the first experimental demonstration that the reaching component of a movement is influenced by number magnitude. Since this paradigm does not require any verbal skill and non-symbolic stimuli (array of dots) can be used, it could be fruitfully adopted to evaluate number abilities in children and even preschoolers. Notably, this is a self-motivating and engaging task, which might help children to get involved and to reduce potential arousal connected to institutional paper-and-pencil examinations.

## Introduction

The propensity to spatially represent environmental information is a core characteristic of human cognitive system (Gevers et al., 2003). Numbers are coded into space along a left-right oriented continuum (Fias and Fischer, 2005; Bueti and Walsh, 2009; Dehaene, 2011). The seminal insight of such a spatial-numerical association goes back to 1880, when Galton (1880) firstly proposed that humans describe and think numbers as increasingly oriented from left to right along a mental number line (MNL), where small numbers are located on the left and large numbers on the right side of space. The first scientific demonstration of this spatial representation of number has been reported more than 100 years later, when Dehaene et al. (1993) discovered that humans respond faster to smaller numbers on the left space and to larger numbers on the right space; the Spatial Numerical Association of Response Codes (SNARC) effect. A large body of literature supports this effect. Humans show a left bias when indicating the center of a string composed of repeated “1”, and a right bias when it is composed of “9” (Fischer, 2001). This indicates that an automatic activation of the left or right space automatically occurs during number processing: the elaboration of small numbers pre-activates the left space and the elaboration of large numerical magnitudes pre-activates the right space.

Complementary results have been obtained in a pseudo-random number generation task. Loetscher et al. (2008) asked participants to report random numbers in the 1–30 numerical range. Participants were systematically influenced by the side (left or right) their head was turned. When they faced toward their left, they produced comparatively more small numbers with respect to when they faced toward their right (Loetscher et al., 2008; see also Winter et al., 2015 for similar results along both near/far space and vertical dimensions, and Hartmann et al., 2012 for a whole body condition). Passive observation of leftward or downward gaze similarly induced participants to generate smaller than large numbers, compared to observing color changes or rightward gaze (Grade et al., 2013). Such biases are explained by a shifting of the attention compatible with the MNL, which facilitates the accessibility to small numbers turning the left and to large numbers turning the right. More recently, the effect reported by Loetscher et al. (2008) has been replicated in a condition of lateral arm turns. The effect was present when two congruent body’s movements were required (e.g., right-turns of both arm and head), but it disappeared whenever the two movements were incongruent (e.g., left-turns of arm and right-turns of head). This reveals that the spatial bias induced by the two sensorimotor locations on numerical processing can annihilate each other (Cheng et al., 2015). All together, these findings show that numbers and motor actions influence each other. This interaction is not limited to laboratory experiences but it emerges also in everyday activities. Numerical magnitude influences directional decisions while walking. In a recent study, healthy adults were required to stand and to produce random numbers as they made lateral turns. Lateral turn decisions could be predicted by the magnitude of random numbers produced before the turn: participants turned left more often when they had just produced small numbers, *vice-versa* they turned right more often when they had just produced large numbers (Shaki and Fischer, 2014).

Since cognitive representations of perceptual and semantic information are fully understood only when considering their impact on actions (Gallese and Lakoff, 2005), the existing knowledge on the MNL should be extended to studies that analyze motor actions while responding to a number (see for example Girelli et al., 2016). An emerging literature of hand-tracking and computer-mouse tracking nicely depict how motor actions can be better understood while performing number related tasks (Song and Nakayama, 2008; Santens et al., 2011; Dotan and Dehaene, 2013; Faulkenberry, 2014; Faulkenberry et al., 2016, 2017).

From this fascinating perspective, adopting kinematic measures is a state-of-art methodology in order to provide a fine-tuned analysis of movement, a large range of degrees of freedom and a highly sensitive investigation. In fact, a mounting number of studies are now using 3-D motion capture and detailed kinematic analyses to measure behavior and to deeply examine questions relating to cognitive processing in naturalistic protocols (for reviews, see Castiello, 2005; Krishnan-Barman et al., 2017). A growing number of studies on prehension movements is proving that semantic information related to magnitude can indeed influence movement kinematics. In particular, it has been shown that grip aperture varies according to the dimension indicated by a label put on a target object: it is larger for the large-labeled object and smaller for the small-labeled object (Gentilucci et al., 2000, 2012; Andres et al., 2008a,b; Namdar et al., 2014). Precision grip movements are faster in response to small numbers and power grips are faster in response to large numbers (Lindemann et al., 2007). These studies clearly show the influence of numbers on motor patterns. However, this effect could also reflect a highly overlearned motor association between magnitude labels (e.g., small, medium, large) and manual responses (e.g., grasping a small or large glass of coke, a 0.5 kg or a 1 kg flour packet). These frequent experiences, though allowing to perform very efficient actions in everyday life (Schwarz and Keus, 2004), could bias to perform smaller grasping actions in relation to smaller digits and vice versa (for review, see Rugani and Sartori, 2016). Notably, two components characterize prehension movements (Jeannerod, 1981; Jakobson and Goodale, 1991; Chieffi and Gentilucci, 1993; Castiello, 1996; Smeets and Brenner, 1999). The reaching component extracts information regarding the object’s spatial location and activates those muscles relevant to approach it. The grasping component extracts information on the object’s intrinsic properties such as size and shape. The open question is whether number processing influences only the grip component or the preceding reaching movement as well (grasp and transport components). To pursue this question in an unbiased way, we recently adopted a new and not-overlearned paradigm (Rugani et al., 2017; see also Betti et al., 2015 for a previous application of this paradigm). We specifically combined a “free response” task with the kinematic analysis of a finger movement and we provided the first demonstration that numerical processing affect not only the grasping, but also the reaching component of movements. This finding particularly depicts the novelty of our approach: instead of measuring the grasping component – which might be affected by previous experience – we adopted a culturally unbiased index (i.e., the transport component). Participants were seated in front of two little soccer goals, one on their left and one on their right side, and they were instructed to kick a small ball with their right index toward the goal indicated by an arrow on the monitor. In a few crucial trials participants were presented also with a small (2) or a large (8) number, and they were allowed to choose the kicking direction. Participants performed more left responses with the small number and more right responses with the large number. The whole kicking movement was then segmented in two temporal phases (i.e., Kick Preparation and Kick Finalization) in order to make a fine-grained analysis of action execution timing. Results showed that in responding to small numbers toward the left and to large numbers toward the right, participants were faster to finalize the action. Moreover, the small number specifically altered the temporal and spatial aspects of left kick’s trajectories, whereas the large number specifically modified right kick’s trajectories. However, a limit of that study is that data concerning the two different movements (i.e., left and right kicks) had to be considered separately due to mechanical and anatomical differences (i.e., the degrees of freedom of the right index finger in relation with the anatomy of the right hand). Here, we adopted a unique action – a straight kick – for all the experimental conditions to avoid any anatomical bias. This means that we expected all the kicks to differ in terms of temporal features of trajectory path, rather than spatial features (i.e., a general leftward deviation was expected across conditions given the degrees of freedom of the right index during the kicking).

Extensive literature on reach-to-grasp consistently showed a general anticipation in hand kinematics when a target object has to be carefully approached (e.g., with the intention to pour vs. to place it, see Schuboe et al., 2008; or with the intention to throw it vs. to lift it, see Armbrüster and Spijkers, 2006). Moreover, it is known that object weight influences motor planning and control of reach-to-grasp actions as to guarantee a stable final grip placement on the object (Weir et al., 1991; Brouwer et al., 2006; Eastough and Edwards, 2007). In particular, Ansuini et al. (2016) recently found that peak velocity between 10 and 40% of normalized movement time was greater when reaching an heavy than a light object. Interestingly, object weight can also influence simply pantomimed reach-to-grasp actions, thus reflecting a link between cognitive representations of the weight and distinctive features of a motor act.

Since the standard parameters utilized for characterizing the reaching component are essentially trajectory and velocity, here we expect that a functional connection between numerical cognition and action planning will translate into different spatial and temporal patterns across conditions. Since numerical priming has two features: (i) spatiality (small numbers are associated with left space and large numbers with right space) and (ii) weight (small numbers are associated with light objects and large numbers with heavy objects), these two features should jointly influence hand movement kinematics. In particular, we predict that the smaller number will influence the temporal aspects of left trajectory deviations, in line with our previous study (Rugani et al., 2017). Whereas the larger number will produce a more direct route, as indexed by lower and shorter trajectory path, and anticipated peak velocity. This innovative approach combining number presentation with action execution with will allow us to measure number-related information transmitted by the hand movements over time.

## Materials and Methods

### Participants

Twenty-three students (10 males and 13 females, mean age = 22.74 years, SD = 0.75) took part in the experiment. A statistical power analysis for sample size estimation was previously performed (GPower 3.1), based on data from a published study (Rugani et al., 2017). The mean effect size (ES) of paired *t*-test in that study (0.65) was considered to be large/medium according to Cohen’s (1988) criteria. Here, since we planned to use a repeated-measure ANOVA, for sample size estimation we inserted these values: η^2^ = 0.20; α = 0.01; 1-β = 0.99; number of measures = 3; groups = 1; supposed correlation among measures = 0.45. The projected sample size needed with this effect size is *N* = 23 for within group comparisons. All participants were right handed, had normal or corrected-to-normal vision, and were naive about the purpose of the experiment. Participants gave their written consent before the experiment. The experimental procedures were approved by the Ethics Committee of the University of Padova and were carried out in accordance with the principles of the 1964 Declaration of Helsinki (Sixth revision, 2008).

### Stimuli

**FIGURE 1:**
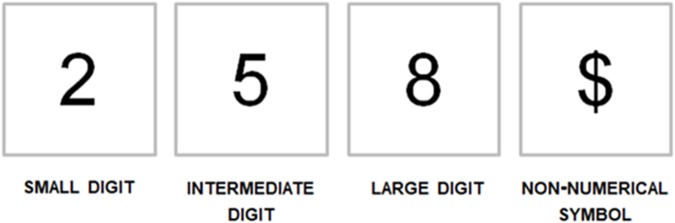
Stimuli adopted in the experiment. A small digit (2), an intermediate digit (5; no-go signal), a large digit (8), and a non-numerical symbol ($).

Stimuli consisted in three symbolic numbers: a small digit (2), an intermediate digit (5), and a large digit (8), plus a symbolic character semantically associated with numbers, though not a number in itself ($, see **Figure 1**). This character was specifically selected on the basis of its symmetry, in order to avoid any indication of direction (as compared to #, for example, which is slightly tilted to the right). The stimulus 5 was adopted as a no-go signal to ensure that reaching movements were not initiated before the number was processed. Hereafter, stimuli will be referred to as S2, S5, S8, and S$. Digits were in Arial font, black color and 160 size. On each trial, a black fixation cross (7.5 cm by 7.5 cm, in Arial font, black color) appeared on the screen before stimulus presentation.

### Apparatus and Procedure

**FIGURE 2:**
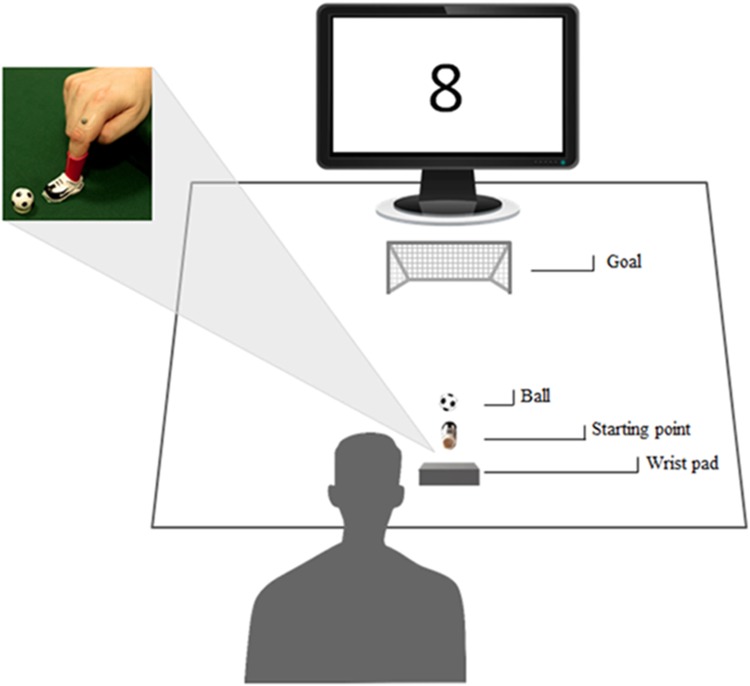
Experimental set up. Participants sat in front of a monitor, wearing with their right index a small soccer shoe positioned on a footprint, in front of a plastic ball. A small goal was placed centrally with respect to the participant’s position. A 3D-Optoelectronic SMART-D system was used to track the kinematics of the participant’s right index finger and the position of the ball by means of six video cameras and two infrared reflective markers taped to the participant’s index finger and to the ball.

Participants sat on a chair in front of a table (90 cm × 90 cm) with the left hand resting on their left leg and the right hand located in the designated start position. The experimental apparatus consisted in a green velvet surface (93.5 cm × 74 cm). Participants’ right index was introduced in the plastic sock (4.5 cm high, 2.5 cm diameter) of a small plastic soccer shoe (3 cm long, 1.5 cm wide; for a schematic representation of the apparatus see **Figure 2**). At the beginning of each trial, participants were instructed to position the shoe on a footprint (3 cm long, 1.5 cm wide) painted on the velvet cloth. A plastic ball (2.3 cm of diameter) was positioned on a plastic ring (1.5 cm diameter) located at 1 cm away from the footprint. In the start position, participants rested their right wrist on a pillow (16 cm long, 11 cm wide and 6.5 cm high), which was shaped to guarantee a comfortable and repeatable posture of the hand, allowing them to effortlessly kick the ball. A small soccer goal (18 cm long 16 cm high) was located 50 cm away from the footprint. A 24” monitor (resolution 1920 × 1080 pixels, refresh frequency 120 Hz) set at eye level (the eye–screen distance was 80 cm) was used to present the experimental stimuli. Participants underwent two sessions (i.e., Training and Testing) and were instructed to kick the ball toward the soccer goal following stimulus presentation, at their own pace. No instruction was given concerning the speed of movement. A black fixation cross appeared for 100 ms and was replaced with a stimulus after 1000 ms. During the Training session S$ was presented for 15 trials. During the Testing session participants kicked the ball upon random presentation of either a symbolic number or a symbolic character: S2, S8, and S$ were shown 10 times each. Whenever S5 was presented (*n* = 10 trials), participants were required to refrain from kicking the ball.

### Kinematic Recording

A 3D-Optoelectronic SMART-D system (Bioengineering Technology and Systems, B|T|S|) was used to track the kinematics of the participant’s right index. One light-weight infrared reflective marker (0.25 mm in diameter; B|T|S|) was taped on the index finger’s proximal phalange to measure the kicking movement (see **Figure 2**). A second marker was located on the ball to compute the midline virtually connecting index finger and target object and to segment the whole movement in a Pre-Contact and a Post-Contact Phase. Six infrared video cameras (sampling rate 140 Hz) detecting the markers’ positions in a 3-D space were placed in a semicircle at a distance of 1–1.2 meters from the table. Each camera position, roll angle, zoom, focus, threshold and brightness were calibrated and adjusted to optimize data collection before each experimental session. For the dynamic calibration, a three-marker wand was moved throughout the workspace of interest for 60 s. The measurements were made along the three Cartesian axes [i.e., *x* (left–right), *y* (up–down), and *z* (anterior–posterior)].The spatial resolution of the recording system was 0.3 mm over the field of view. The standard deviation of the reconstruction error was 0.2 mm for the *x*, *y*, and *z* axes.

### Data Processing

Following kinematic data collection, the SMART-D Tracker software package (B|T|S|) was used to provide a 3-D reconstruction of the marker positions of each trial as a function of time. The data were then filtered using a finite impulse response linear filter (transition band = 1 Hz, sharpening variable = 2, cut-off frequency = 10 Hz; D’Amico and Ferrigno, 1990, 1992). Movement onset was defined as the time at which the tangential velocity of the finger marker crossed a threshold (5 mm/s) and remained above it for longer than 500 ms. End of movement was defined as the time at which the tangential velocity of the finger marker dropped below the threshold (5 mm/s) after the ball was kicked. The following kinematic parameters were extracted for each individual movement using a custom Protocol run in Matlab, 2014b (The 4 Math Works, Natick, MA, United States):

*Movement Time*: the time interval between movement onset and end of movement (ms);

*Trajectory Path*: the length of the index trajectory (mm);

*Maximum Trajectory Height:* the maximum height of the index trajectory on the y-axis (mm);

*Contact Time*: the time at which the tangential velocity of the ball crossed a threshold (2 mm/s) and remained above it for longer than 500 ms;

*Time to Maximum Velocity*: the time at which index velocity was maximum, with respect to movement onset (ms);

*Time to Maximum Trajectory Height*: the time at which index trajectory was higher, with respect to movement onset (ms);

*Time to Maximum Trajectory Deviation*: the time at which index trajectory reached the maximum perpendicular deviation from the virtual line linking the starting position with the target object, with respect to movement onset (ms).

The temporal peaks were then normalized with respect to movement time, so that individual speed differences were accounted for:

*Contact Time (%)*: the percentage of movement time at which the tangential velocity of the ball crossed a threshold (2 mm/s) and remained above it for longer than 500 ms;

*Time of Maximum Velocity (%)*: the percentage of movement time at which the index trajectory was at maximum velocity (%);

*Time to Maximum Trajectory Height (%)*: the percentage of movement time at which the index trajectory reached its higher peak (%).

*Time to Maximum Trajectory Deviation (%)*: the percentage of movement time at which index trajectory reached the maximum deviation from the midline (%).

For each participant and kinematic index, we calculated means and relative standard deviations for each type of stimulus (S2, S8, and S$).

### Data Analysis

The mean values for each parameter of interest were determined for each participant and entered into separate repeated-measures ANOVAs with Stimulus (S2, S8, and S$) as within-subjects factor.

Preliminary analyses were conducted to check for normality, sphericity (Mauchly test), univariate and multivariate outliers, with no violations noted. For the ANOVA the alpha level of p was set <0.01, in accordance with our power analysis. Main effects were used to explore the means of interest (*post hoc t*-test) and Bonferroni correction was applied (alpha level of *p* < 0.05) to prevent Type-1 errors. Statistical analyses were performed with SPSS 23 (SPSS Inc., Chicago, IL, United States) software.

## Results

All the means, medians and standard errors are summarized in **Table 1**.

**Table 1:** Statistically significant key kinematic parameters (mean, standard errors, and median per condition) across stimuli.

	S2	S8	$
*Movement time (ms)*	370.30 (± 20.28)	341. 12 (± 20.11)	359.45 (± 18.39)
	376.67	351.52	358.33
*Trajectory path (mm)*	38.58 (± 4.16)	34.95 (± 3.84)	37.63 (± 4.16)
	34.21	31.41	34.25
*Maximum trajectory height (mm)*	95.96 (± 2.11)	87.07 (± 1.85)	95.61 (± 1.91)
	94.76	86.97	94.88
*Time to maximum velocity (%)*	65 (± 03)	56 (± 03)	64 (± 03)
	66	59	64
*Time to maximum trajectory*	84 (± 04)	77 (± 04)	84 (± 04)
*height (%)*	94	85	95
*Time to maximum trajectory*	51 (± 03)	44 (± 02)	48 (± 02)
*deviation (%)*	52	42	52

*Movement Time (ms):* The ANOVA performed on MT revealed a non-significant effect of Stimulus [*F*_(2,44)_ = 3.48, *p* = 0.04, $\eta_{p}^{2}$ = 0.14].

*Trajectory Path (mm)*: The ANOVA performed on the length of the index trajectory revealed a significant effect of Stimulus [*F*_(2,44)_ = 4.75, *p* = 0.01, $\eta_{p}^{2}$ = 0.18]. Observing S8 led to a shorter trajectory with respect to observing S2 (*p* = 0.01). This effect was significant also for S8 compared to S$ (*p* = 0.02).

*Maximum Trajectory Height (mm):* The ANOVA performed on the maximum height of the index trajectory revealed a significant effect of Stimulus [*F*_(2,44)_ = 323.98, *p* < 0.001, $\eta_{p}^{2}$ = 0.94]. Observing S8 led to a lower trajectory with respect to observing S2 (*p* < 0.001). This effect was significant also for S8 compared to S$ (*p* < 0.001).

*Contact Time (%):* The ANOVA performed on CT revealed a non-significant effect of Stimulus [*F*_(2,44)_ = 0.55, *p* = 0.58, $\eta_{p}^{2}$ = 0.02].

*Time to Maximum Velocity (%):* The ANOVA performed on the time at which index velocity was maximum revealed a significant effect of Stimulus [*F*_(2,44)_ = 13.35, *p* < 0.001, $\eta_{p}^{2}$ = 0.38]. Observing S8 led to an earlier peak with respect to observing S2 (*p* < 0.001). This effect was significant also for S8 compared to S$ (*p* < 0.001).

*Time to Maximum Trajectory Height (%)*: The ANOVA performed on the time at which index trajectory was higher revealed a significant effect of Stimulus [*F*_(2,44)_ = 9.07, *p* < 0.001, $\eta_{p}^{2}$ = 0.29]. Observing S8 led to an earlier peak with respect to observing S2 (*p* < 0.001). This effect was significant also for S8 compared to S$ (*p* = 0.02).

**FIGURE 3:**
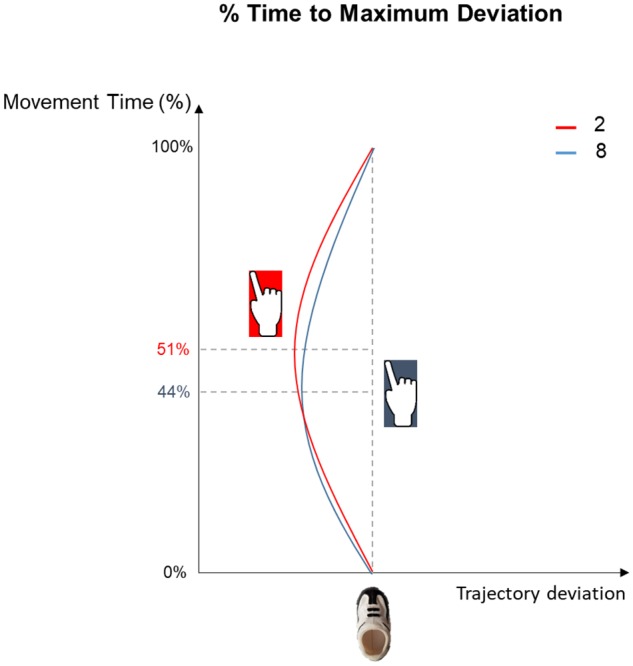
Graphical representation of % of Time to Maximum Deviation results. Leftward trajectory deviations following S2 and S8 presentation (red and blue lines, respectively) show a delayed peak for the small number presentation (51%) compared to the larger number (44%).

*Time to Maximum Trajectory Deviation (%):* The ANOVA performed on the time at which index trajectory reached the maximum deviation from the midline revealed a significant effect of Stimulus [*F*_(2,44)_ = 9.07, *p* < 0.001, $\eta_{p}^{2}$ = 0.29]. Observing S2 led to a delayed leftward deviation with respect to observing S8 (*p* < 0.001; see **Figure 3**).

## Discussion

The aim of this study was to determine whether number processing affects the performance of executed movements. Participants were asked to perform a kicking action with their right hand after observing a small/large digit (2, 8) or a symbolic character ($). Our main finding is that although executed actions were exactly the same across conditions, a decrease in Trajectory Path, Trajectory Height, Time to Maximum Velocity and Time to Maximum Trajectory Height occurred for the large compared to the small digit. Our results are in line with previous studies demonstrating a general anticipation when an object is approached more carefully (e.g., Armbrüster and Spijkers, 2006; Schuboe et al., 2008) and an early velocity peak when reaching a heavy than a light object (Ansuini et al., 2016). In our study, performing a finger kicking action after observation of a large digit was indeed highly efficient: the trajectory path was shorter and lower on the surface and the velocity peak was anticipated. In particular, we found an anticipation of the Time of Maximum Velocity ranging from S8 (56%) to S$ (64%) and S2 (65%), despite the executed movement was the same. Since a statistically significant effect on Time to Maximum Velocity was specifically connected to the observation of S8, this might suggests the activation of an association between larger numbers and weight. By combining knowledge regarding numerical magnitude and weight dynamics, the motor system might be able to adjust kick kinematics accordingly.

A crucial data arising from the present data is the temporal aspect of trajectory deviations. Given the very short distance between footprint and ball (1 cm) and the constrained end-goal (i.e., straight kick), no effect was expected in terms of trajectory deviations before contact. However, a longer tilt leftwards for the S2 condition during the post-contact phase seems to indicate that participants were aiming towards the left following small number presentation compared to large number presentation. Similar results were obtained with an index finger pointing task (Fischer, 2003). Participants were faster when pointing leftward after a small digit presentation and rightward after a large digit presentation (Fischer, 2003). Subsequent studies in adults (Ishihara et al., 2006) and in 7 year-old children (Möhring et al., 2017) revealed that this bias could be explained by a contamination of motor preparation by a direct activation of number magnitude whit the congruent spatial location.

Our data show for the first time that the control mechanisms underlying reaching formation are affected by number processing beyond the – already demonstrated – grasping component. Since the effect of numerical magnitude on grip aperture kinematics (Gentilucci et al., 2000, 2012; Andres et al., 2004, 2008a,b; Lindemann et al., 2007; Moretto and di Pellegrino, 2008; Chiou et al., 2012; Namdar et al., 2014) and object affordances (Badets et al., 2007; Chiou et al., 2009) is well known, the present data significantly extend previous literature. The impact of numerical magnitude on both reaching and grasping kinematics would corroborate the theory that representations of number and actions share common codes within a magnitude representation’s system (Lindemann et al., 2007).

### The ATOM Theory and Numerical Affordance

From a neuropsychological viewpoint, the modulation of numerical cognition on action control could be explained by the “A Theory of Magnitude”, or “ATOM theory” (Walsh, 2003; see also Bueti and Walsh, 2009 for an updated proposal). The ATOM theory postulates that the intra-parietal sulcus (IPS) serves as the cortical center for time, space, and numbers estimation. The IPS would be equipped with an analog system that constantly computes magnitudes for action execution (Bueti and Walsh, 2009). It is therefore plausible that beyond object affordances related to the physical features of an object (Gibson, 1979; Tucker and Ellis, 1998), a “*numerical affordance*” might link objects’ extension and numerousness to specific motor dynamics. Here we specifically demonstrated that two features related to numbers (spatiality and weight) are interrelated and affect movement kinematics. From this perspective, the ATOM theory may explain the interaction between numerical information and non-numerical magnitude, such as time and space, and especially how numbers prompt spatially oriented actions (SNARC effect).

In neural terms, reaching and grasping components are mediated by two separate anatomical pathways (for review see Filimon, 2010). Grasping is organized by a lateral parieto-frontal circuit and reaching by a more medial parieto-frontal circuit including medial intraparietal area and dorsal premotor area (Filimon, 2010; Di Bono et al., 2015). Notably, the MLN is linked to a parietal network: Consistent with the ATOM theory (Walsh, 2003; Bueti and Walsh, 2009), the brain regions dedicated to number processing and to reach-to-grasp movements are closely linked by a generalized magnitude system, which transforms quantitative information into actions. In this connection, it would be important to consider the neural mechanism linking number and reaching movement. Functional neuroimaging studies will help to clarify the differential contribution of the reaching and the grasping components to number processing in action execution.

### Embodied Number Processing

Our evidence, highlighting spatial and temporal properties of finger movements responding to numbers, fits with the embodied theory of numerical representation. From this perspective, numbers are not abstract, but embodied, i.e., rooted in bodily experiences. The way in which we use our bodies to act can influence our cognition (for an overview see Wilson, 2002; Barsalou, 2008; Patro et al., 2015). For example, the sensory-motor activations which occur during learning shape the newly learnt representation (Fischer and Zwaan, 2008; Fischer, 2012). Since number acquisition usually implies concomitant body-movements, like finger counting (Brissiaud, 1992; Domahs et al., 2010), such embodied space-motor-number relations have also been used for training. For example it has been shown that playing games eliciting an embodied experience of the spatial layout of the MNL improves numerical competences (Ramani and Siegler, 2008; Whyte and Bull, 2008; Siegler and Ramani, 2009).

The challenging perspective of embodied cognition offers a stimulating approach to the study of mathematical competences. The analysis of finger movements indeed is considered a powerful method to assess numerical representations (Dotan and Dehaene, 2013). Paradigms focused on finger trajectories could be used to assess mental computations, and might offer a diagnostic instrument for measuring both normal and pathological development of mathematical competences (Booth and Siegler, 2006).

## Conclusion

This study aimed at deepens our knowledge on the link between spatial numerical association and action execution. From an evolutive perspective, it could extend existing evidence on the origin of the spatial numerical association (de Hevia and Spelke, 2009; de Hevia et al., 2012; McCrink and Opfer, 2014; Nuerk et al., 2015; Rugani and de Hevia, 2016; Möhring et al., 2017). Moreover, it could allow to clarify whether and how symbolic and non-symbolic numbers (see Rugani et al., 2017 for a definition) affect the sensorimotor transformations related to the motor control of the hand. This is particularly relevant considering the role of finger counting in number processing (Di Luca et al., 2006; Fischer, 2008; Sixtus et al., 2017), which survives in adults and seems to help the associations between numbers and hand actions (Hatano et al., 1977; Hubbard et al., 2005). The important relation between finger counting and mathematical abilities is scientifically documented. Abacus experts spontaneously move their hands while solving arithmetic calculation (Hatano et al., 1977). The manumerical cognition hypothesis (Hubbard et al., 2005) claims that this relation could explicate why dyscalculia, left–right confusion and finger agnosia often co-occur in the Gerstmann syndrome.

Last but not least, our protocol – based on a self-motivating and engaging task – would allow to investigate numerical cognition and its relation with space without the anxiety which usually disadvantages children with problems in mathematical comprehension (for the use of an innovative approach on mathematical learning with Touchscreen Tablets, see Duijzer et al., 2017). Highly math-anxious persons have a common and strong tendency to avoid math, which reduces their possibility to increase their math competences (Ashcraft, 2002).

This paradigm, characterized by a new and not-overlearned task, could therefore be used to study the relation between number processing and motor action over development, before and during mathematical learning. Movements kinematics not only provides an accurate measure of the association between numbers and actions, but could also offer a novel tool for the diagnosis of potential mathematical deficits.

## Author Contributions

RR and LS designed the study, created the stimuli, collected, analyzed the data, and wrote the manuscript. LS did the statistical analyses. RR, SB, and LS interpreted the data discussed the results. SB created the figures.

## Conflict of Interest Statement

The authors declare that the research was conducted in the absence of any commercial or financial relationships that could be construed as a potential conflict of interest.
